# Supplementary material for: Genome Investigation and Functional Annotation of Lactiplantibacillus plantarum YW11 Revealing Streptin and Ruminococcin-A as Potent Nutritive Bacteriocins against Gut Symbiotic Pathogens
Source: Molecules. 2023 Jan 4;28(2):491. doi: 10.3390/molecules28020491 (PMC9862464; doi:10.3390/molecules28020491)
Supplement: Supplementary file 1 [file molecules-28-00491-s001.zip › molecules-2009576-Supplementary file-2nd proof.pdf]

A.

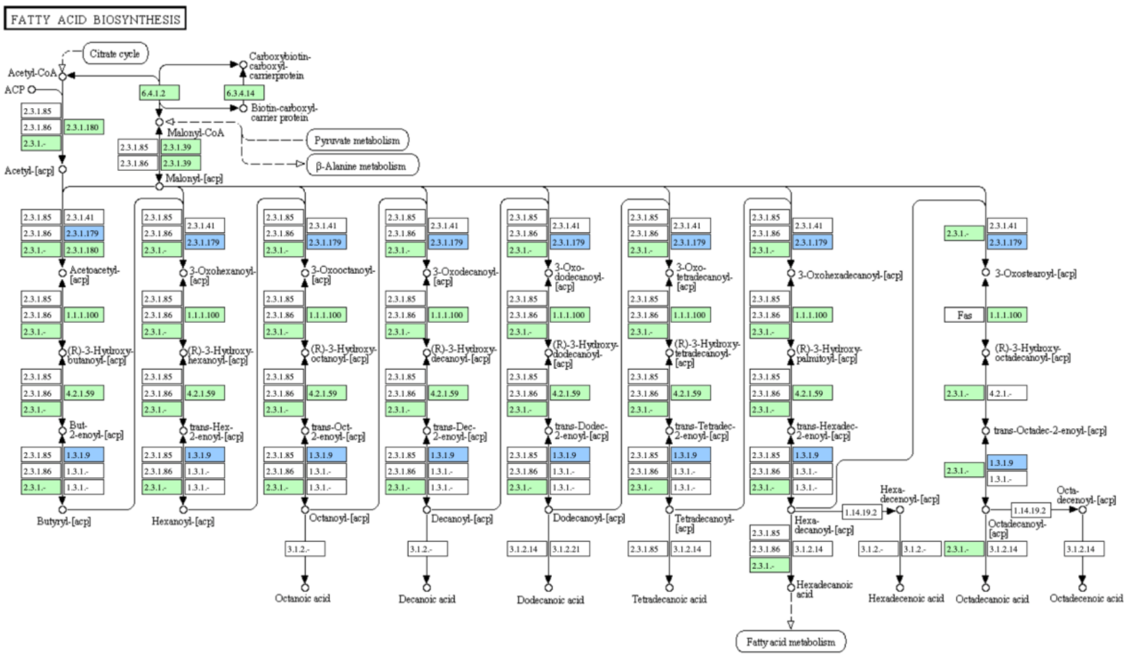
FATTY ACID BIOSYNTHESIS
Citrate cycle
Acetyl-CoA
ACP
Carboxybiotin-carboxyl-carrierprotein
Biotin-carboxyl-carrier protein
Malonyl-CoA
Pyruvate metabolism
β-Alanine metabolism
Acetyl-[acp]
Malonyl-[acp]
Butyryl-[acp]
Hexanoyl-[acp]
Octanoyl-[acp]
Decanoyl-[acp]
Dodecanoyl-[acp]
Tetradecanoyl-[acp]
Hexadecanoic acid
Octanoic acid
Decanoic acid
Dodecanoic acid
Tetradecanoic acid
Hexadecenoic acid
Octadecanoic acid
Octadecenoic acid
Fatty acid metabolism

B.

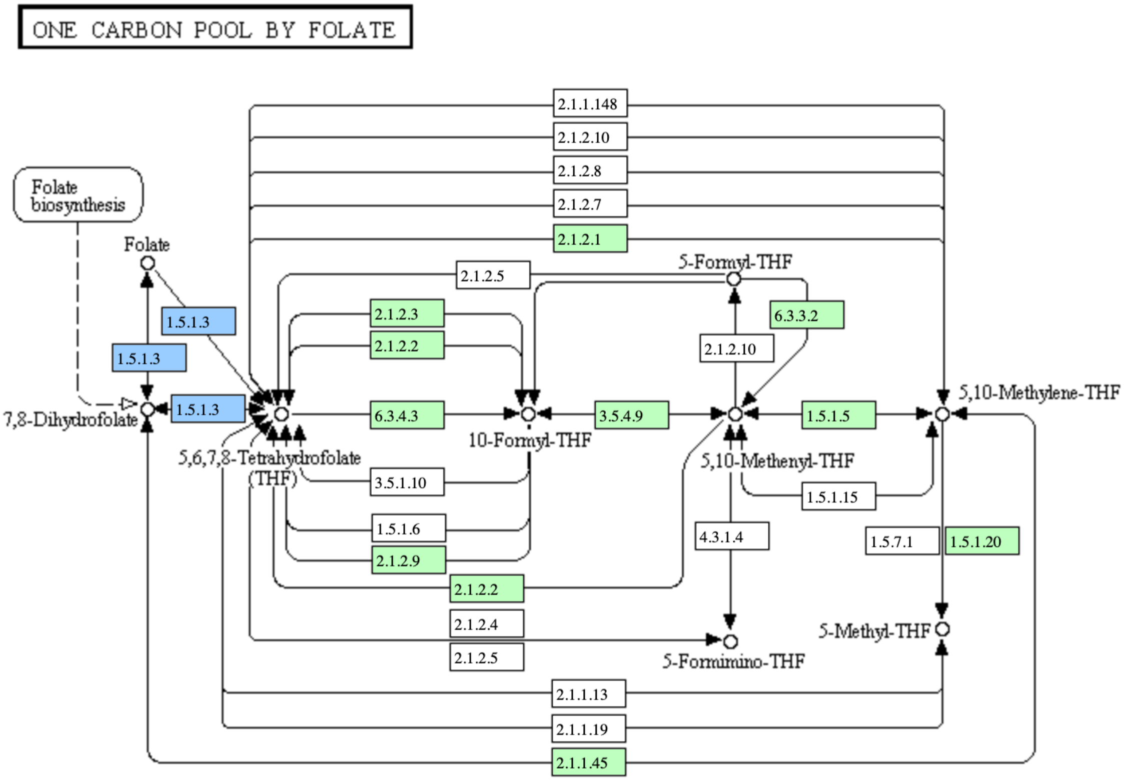
ONE CARBON POOL BY FOLATE
2.1.1.148
2.1.2.10
2.1.2.8
2.1.2.7
2.1.2.1
Folate biosynthesis
Folate
2.1.2.5
5-Formyl-THF
2.1.2.3
6.3.3.2
1.5.1.3
2.1.2.2
1.5.1.3
2.1.2.10
1.5.1.3
6.3.4.3
3.5.4.9
1.5.1.5
5,10-Methylene-THF
7,8-Dihydrofolate
10-Formyl-THF
5,6,7,8-Tetrahydrofolate (THF)
5,10-Methenyl-THF
3.5.1.10
1.5.1.15
1.5.1.6
4.3.1.4
1.5.7.1
1.5.1.20
2.1.2.9
2.1.2.2
2.1.2.4
5-Methyl-THF
2.1.2.5
5-Formimino-THF
2.1.1.13
2.1.1.19
2.1.1.45

C.

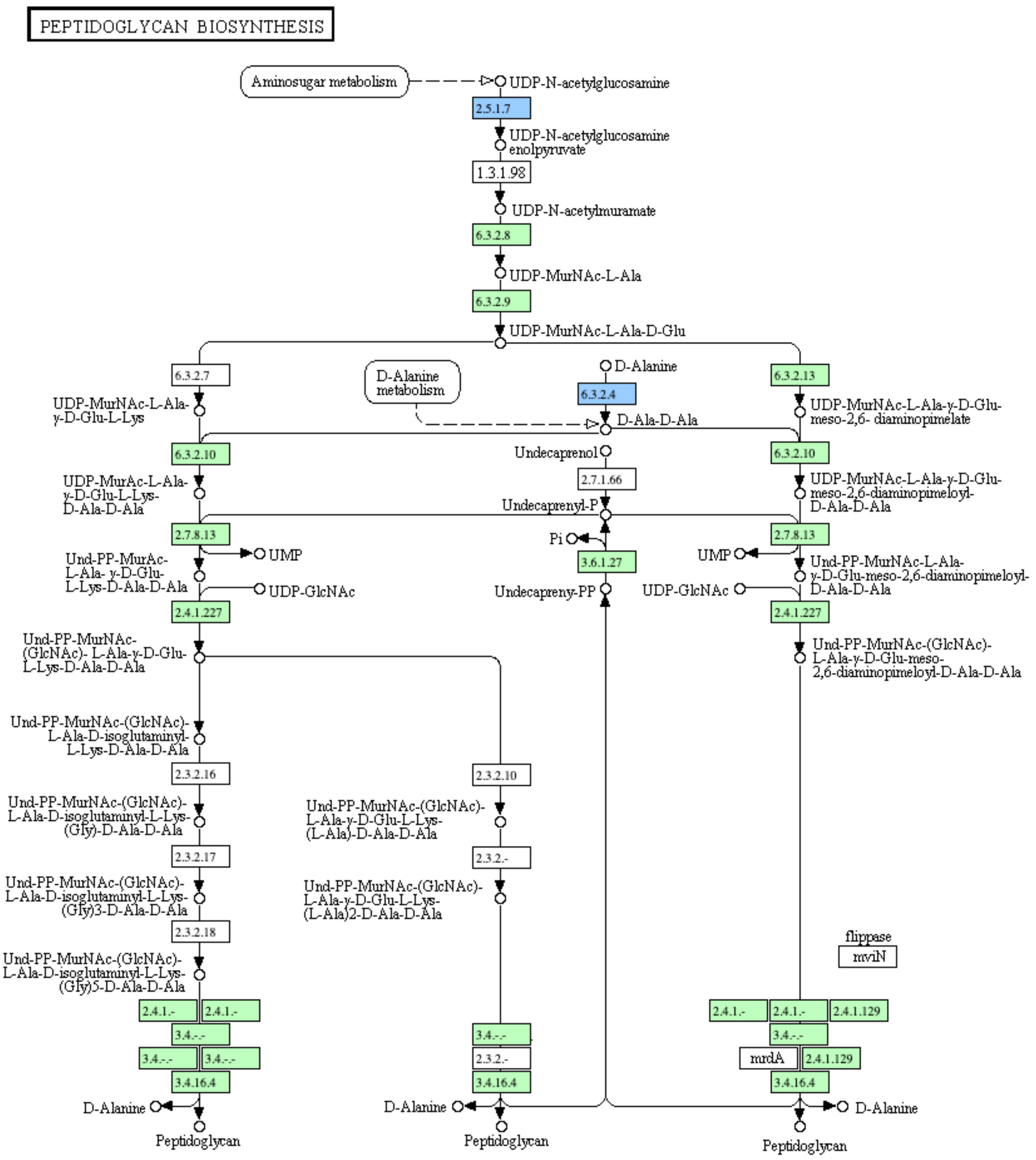

PEPTIDOGLYCAN BIOSYNTHESIS
Aminosugar metabolism
UDP-N-acetylglucosamine
2.5.1.7
UDP-N-acetylglucosamine enolpyruvate
1.3.1.98
UDP-N-acetylmuramate
6.3.2.8
UDP-MurNAc-L-Ala
6.3.2.9
UDP-MurNAc-L-Ala-D-Glu
6.3.2.7
UDP-MurNAc-L-Ala-γ-D-Glu-L-Lys
D-Alanine metabolism
D-Alanine
6.3.2.4
D-Ala-D-Ala
6.3.2.13
UDP-MurNAc-L-Ala-γ-D-Glu-meso-2,6- diaminopimelate
6.3.2.10
UDP-MurAc-L-Ala-γ-D-Glu-L-Lys-D-Ala-D-Ala
Undecaprenol
2.7.1.66
Undecaprenyl-P
UDP-MurNAc-L-Ala-γ-D-Glu-meso-2,6-diaminopimeloyl-D-Ala-D-Ala
2.7.8.13
UMP
Pi
3.6.1.27
Und-PP-MurAc-L-Ala- γ-D-Glu-L-Lys-D-Ala-D-Ala
UDP-GlcNAc
Undecapreny-PP
Und-PP-MurNAc-L-Ala-γ-D-Glu-meso-2,6-diaminopimeloyl-D-Ala-D-Ala
2.4.1.227
Und-PP-MurNAc-(GlcNAc)- L-Ala-γ-D-Glu-L-Lys-D-Ala-D-Ala
Und-PP-MurNAc-(GlcNAc)-L-Ala-γ-D-Glu-meso-2,6-diaminopimeloyl-D-Ala-D-Ala
Und-PP-MurNAc-(GlcNAc)-L-Ala-D-isoglutaminyl-L-Lys-D-Ala-D-Ala
2.3.2.16
2.3.2.10
Und-PP-MurNAc-(GlcNAc)-L-Ala-D-isoglutaminyl-L-Lys-(Gly)-D-Ala-D-Ala
Und-PP-MurNAc-(GlcNAc)-L-Ala-γ-D-Glu-L-Lys-(L-Ala)-D-Ala-D-Ala
2.3.2.17
2.3.2.-
Und-PP-MurNAc-(GlcNAc)-L-Ala-D-isoglutaminyl-L-Lys-(Gly)3-D-Ala-D-Ala
Und-PP-MurNAc-(GlcNAc)-L-Ala-γ-D-Glu-L-Lys-(L-Ala)2-D-Ala-D-Ala
2.3.2.18
Und-PP-MurNAc-(GlcNAc)-L-Ala-D-isoglutaminyl-L-Lys-(Gly)5-D-Ala-D-Ala
flippase
mviN
2.4.1.-
2.4.1.129
3.4.-.-
mrdA
3.4.16.4
D-Alanine
Peptidoglycan

D.

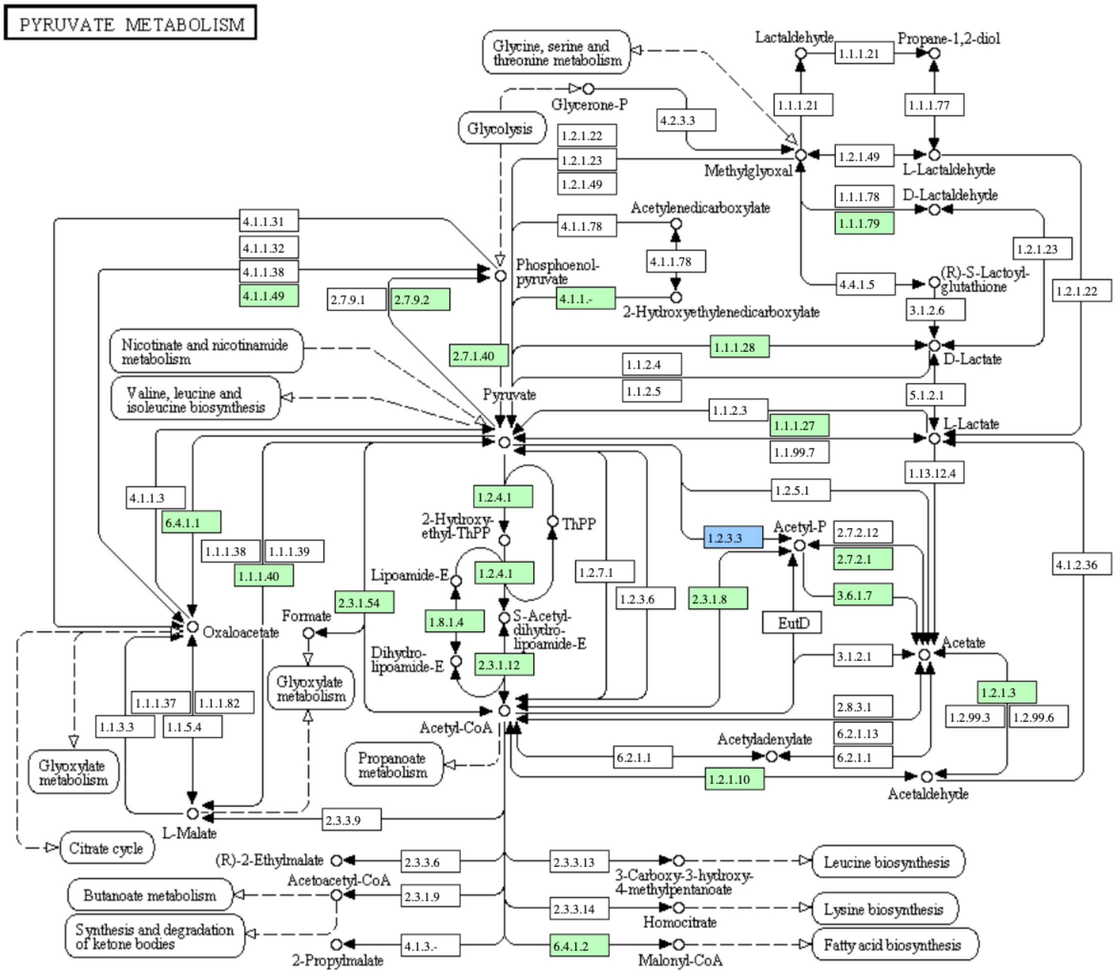
PYRUVATE METABOLISM
Glycine, serine and threonine metabolism
Lactaldehyde
Propane-1,2-diol
1.1.1.21
1.1.1.77
Glycerone-P
4.2.3.3
Glycolysis
1.2.1.22
1.2.1.23
1.2.1.49
Methylglyoxal
L-Lactaldehyde
1.1.1.78
D-Lactaldehyde
1.1.1.79
Acetylenedicarboxylate
4.1.1.78
4.1.1.31
4.1.1.32
4.1.1.38
4.1.1.49
Phosphoenol-pyruvate
2.7.9.1
2.7.9.2
4.1.1.-
2-Hydroxyethylenedicarboxylate
4.4.1.5
(R)-S-Lactoyl-glutathione
3.1.2.6
1.2.1.23
1.2.1.22
Nicotinate and nicotinamide metabolism
Valine, leucine and isoleucine biosynthesis
2.7.1.40
1.1.1.28
1.1.2.4
1.1.2.5
D-Lactate
5.1.2.1
Pyruvate
1.1.2.3
1.1.1.27
1.1.99.7
L-Lactate
1.13.12.4
4.1.1.3
6.4.1.1
1.2.4.1
2-Hydroxy-ethyl-ThPP
ThPP
1.2.5.1
1.2.3.3
Acetyl-P
2.7.2.12
2.7.2.1
3.6.1.7
4.1.2.36
1.1.1.38
1.1.1.39
1.1.1.40
Lipoamide-E
1.2.7.1
1.2.3.6
2.3.1.8
EutD
2.3.1.54
Formate
1.8.1.4
S-Acetyl-dihydro-lipoamide-E
Oxaloacetate
Dihydro-lipoamide-E
2.3.1.12
3.1.2.1
Acetate
Glyoxylate metabolism
1.2.1.3
1.2.99.3
1.2.99.6
1.1.1.37
1.1.1.82
2.8.3.1
1.1.3.3
1.1.5.4
Acetyl-CoA
6.2.1.13
Propanoate metabolism
Acetyladenylate
Glyoxylate metabolism
6.2.1.1
6.2.1.1
1.2.1.10
Acetaldehyde
L-Malate
2.3.3.9
Citrate cycle
(R)-2-Ethylmalate
2.3.3.6
2.3.3.13
3-Carboxy-3-hydroxy-4-methylpentanoate
Leucine biosynthesis
Acetoacetyl-CoA
Butanoate metabolism
2.3.1.9
2.3.3.14
Homocitrate
Lysine biosynthesis
Synthesis and degradation of ketone bodies
2-Propylmalate
4.1.3.-
6.4.1.2
Malonyl-CoA
Fatty acid biosynthesis

E.

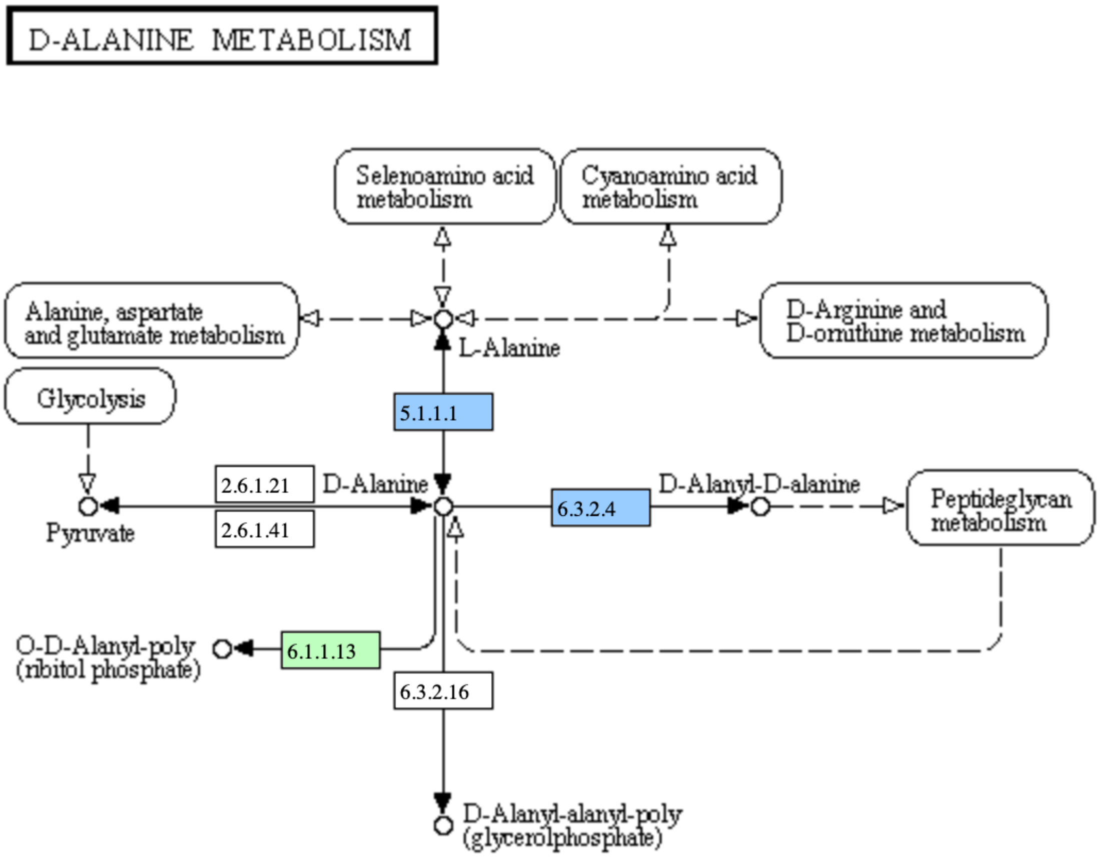
D-ALANINE METABOLISM
Selenoamino acid metabolism
Cyanoamino acid metabolism
Alanine, aspartate and glutamate metabolism
D-Arginine and D-ornithine metabolism
L-Alanine
Glycolysis
5.1.1.1
2.6.1.21
2.6.1.41
D-Alanine
D-Alanyl-D-alanine
6.3.2.4
Peptideglycan metabolism
Pyruvate
O-D-Alanyl-poly (ribitol phosphate)
6.1.1.13
6.3.2.16
D-Alanyl-alanyl-poly (glycerolphosphate)

F.

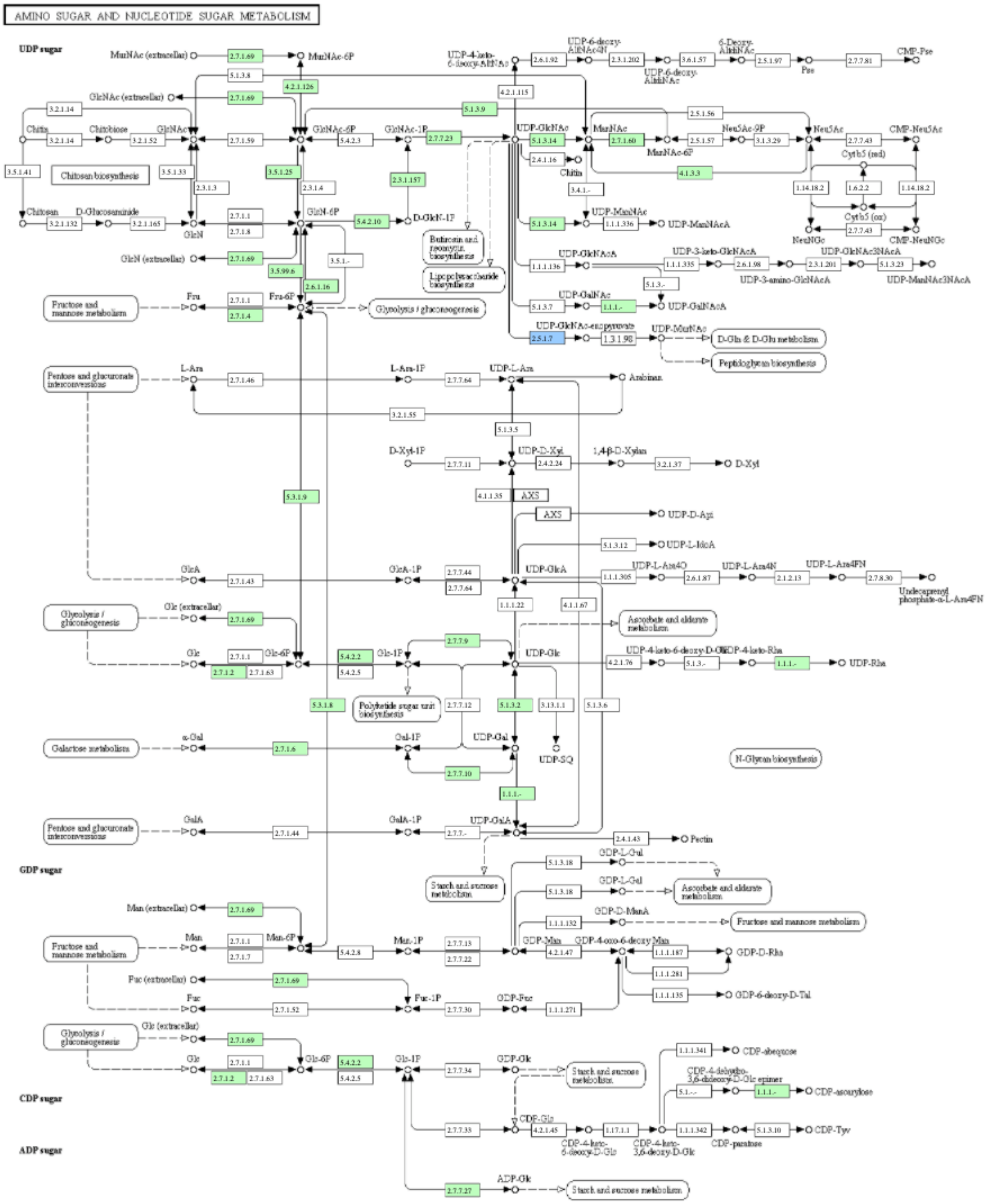
AMINO SUGAR AND NUCLEOTIDE SUGAR METABOLISM
UDP sugar
GDP sugar
CDP sugar
ADP sugar

G.

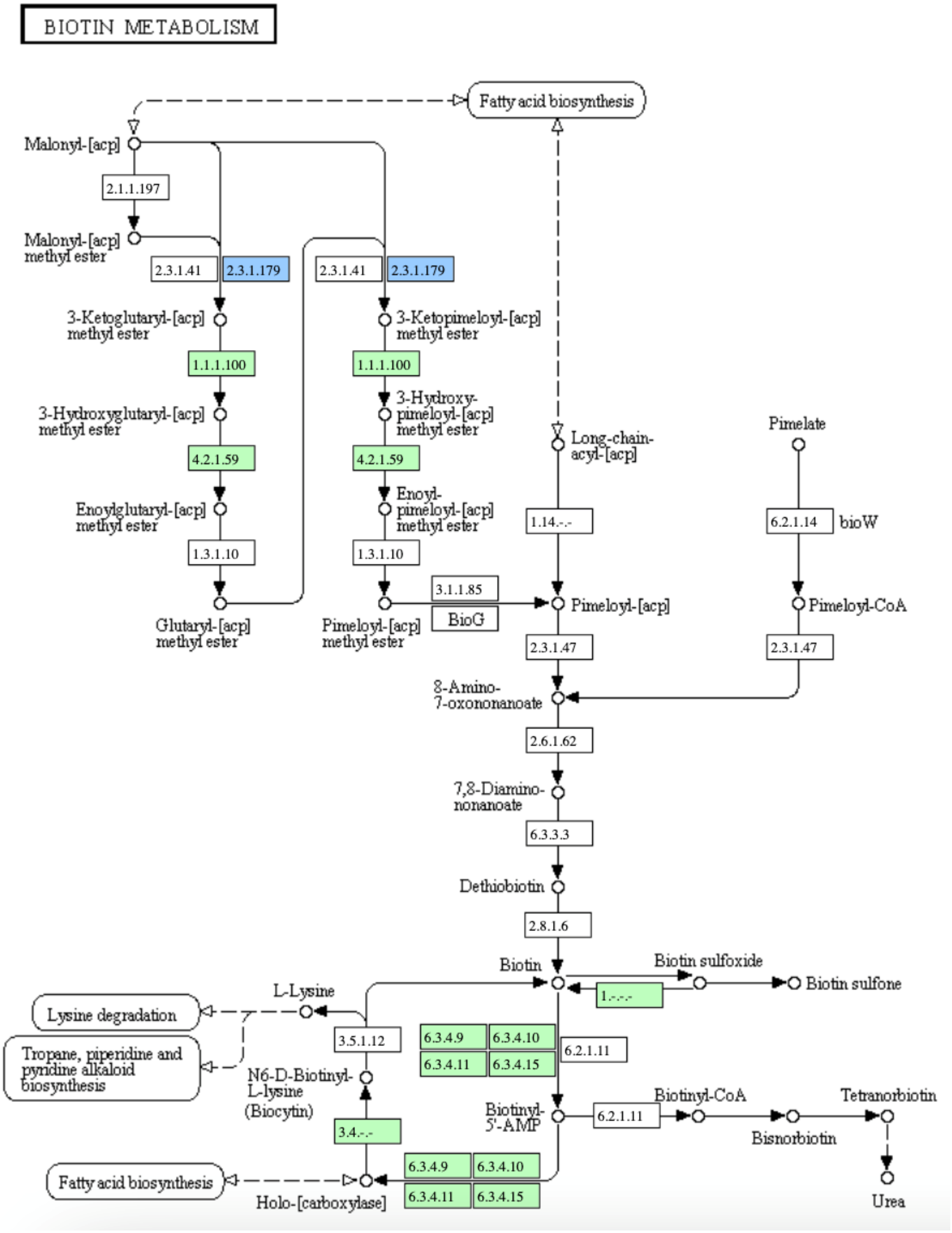

BIOTIN METABOLISM
Fatty acid biosynthesis
Malonyl-[acp]
2.1.1.197
Malonyl-[acp] methyl ester
2.3.1.41
2.3.1.179
2.3.1.41
2.3.1.179
3-Ketoglutaryl-[acp] methyl ester
3-Ketopimeloyl-[acp] methyl ester
1.1.1.100
1.1.1.100
3-Hydroxyglutaryl-[acp] methyl ester
3-Hydroxy-pimeloyl-[acp] methyl ester
4.2.1.59
4.2.1.59
Enoylglutaryl-[acp] methyl ester
Enoyl-pimeloyl-[acp] methyl ester
1.3.1.10
1.3.1.10
Glutaryl-[acp] methyl ester
Pimeloyl-[acp] methyl ester
3.1.1.85
BioG
Long-chain-acyl-[acp]
1.14.-.-
Pimelate
6.2.1.14
bioW
Pimeloyl-[acp]
Pimeloyl-CoA
2.3.1.47
2.3.1.47
8-Amino-7-oxononanoate
2.6.1.62
7,8-Diamino-nonanoate
6.3.3.3
Dethiobiotin
2.8.1.6
Biotin
Biotin sulfoxide
Biotin sulfone
1.-.-.-
L-Lysine
Lysine degradation
Tropane, piperidine and pyridine alkaloid biosynthesis
3.5.1.12
6.3.4.9
6.3.4.10
6.3.4.11
6.3.4.15
6.2.1.11
N6-D-Biotinyl-L-lysine (Biocytin)
3.4.-.-
Biotinyl-5'-AMP
6.2.1.11
Biotinyl-CoA
Bisnorbiotin
Tetranorbiotin
Urea
Fatty acid biosynthesis
Holo-[carboxylase]
6.3.4.9
6.3.4.10
6.3.4.11
6.3.4.15

H.

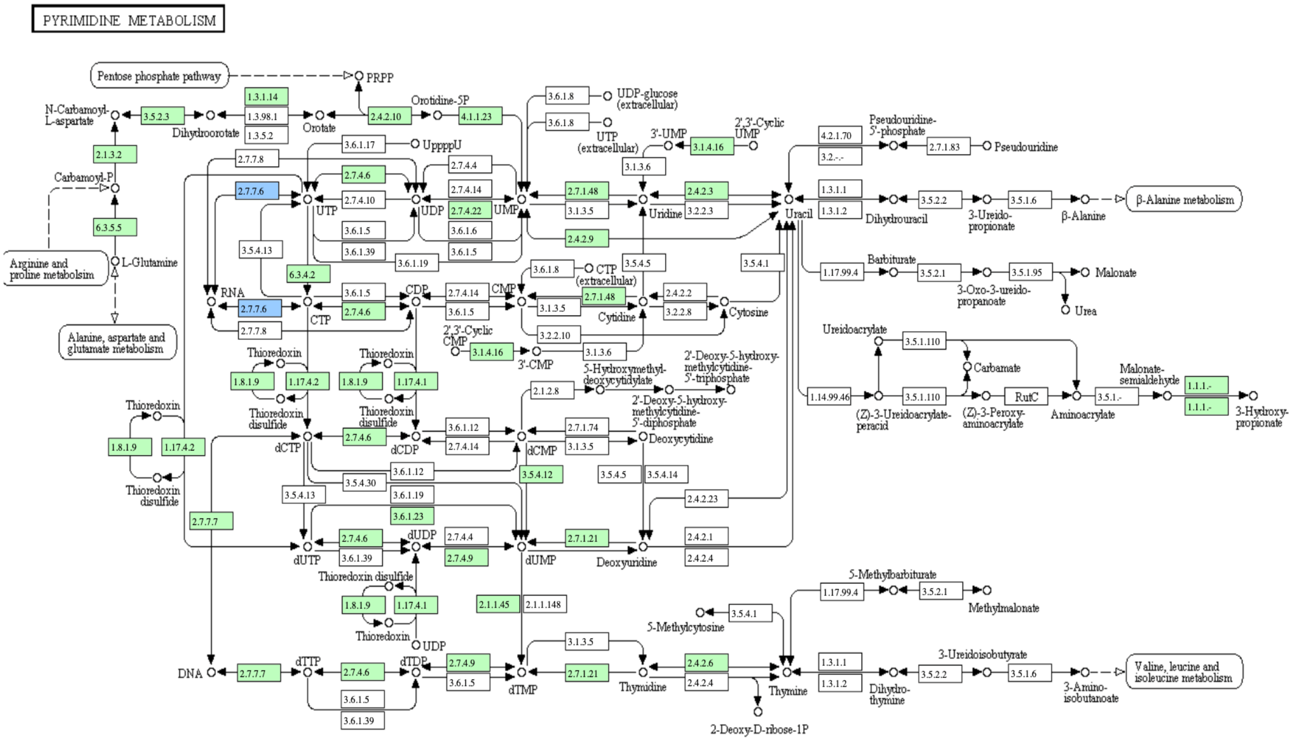
PYRIMIDINE METABOLISM
Pentose phosphate pathway
PRPP
N-Carbamoyl-L-aspartate
Dihydroorotate
Orotate
Orotidine-5P
UDP-glucose (extracellular)
UTP (extracellular)
3'-UMP
2',3'-Cyclic UMP
UppppU
Carbamoyl-P
L-Glutamine
Arginine and proline metabolsim
Alanine, aspartate and glutamate metabolism
UTP
UDP
UMP
Uridine
RNA
CTP
CDP
CMP
Cytidine
Cytosine
CTP (extracellular)
2',3'-Cyclic CMP
3'-CMP
Thioredoxin
Thioredoxin disulfide
dCTP
dCDP
dCMP
Deoxycytidine
5-Hydroxymethyl-deoxycytidylate
2'-Deoxy-5-hydroxy-methylcytidine-5'-diphosphate
2'-Deoxy-5-hydroxy-methylcytidine-5'-triphosphate
dUTP
dUDP
dUMP
Deoxyuridine
UDP
DNA
dTTP
dTDP
dTMP
Thymidine
Thymine
5-Methylcytosine
2-Deoxy-D-ribose-1P
Uracil
Pseudouridine-5'-phosphate
Pseudouridine
Dihydrouracil
3-Ureido-propionate
β-Alanine
β-Alanine metabolism
Barbiturate
3-Oxo-3-ureido-propanoate
Malonate
Urea
Ureidoacrylate
Carbamate
(Z)-3-Ureidoacrylate-peracid
(Z)-3-Peroxy-aminoacrylate
RutC
Aminoacrylate
Malonate-semialdehyde
3-Hydroxy-propionate
5-Methylbarbiturate
Methylmalonate
Dihydro-thymine
3-Ureidoisobutyrate
3-Amino-isobutanoate
Valine, leucine and isoleucine metabolism

I.

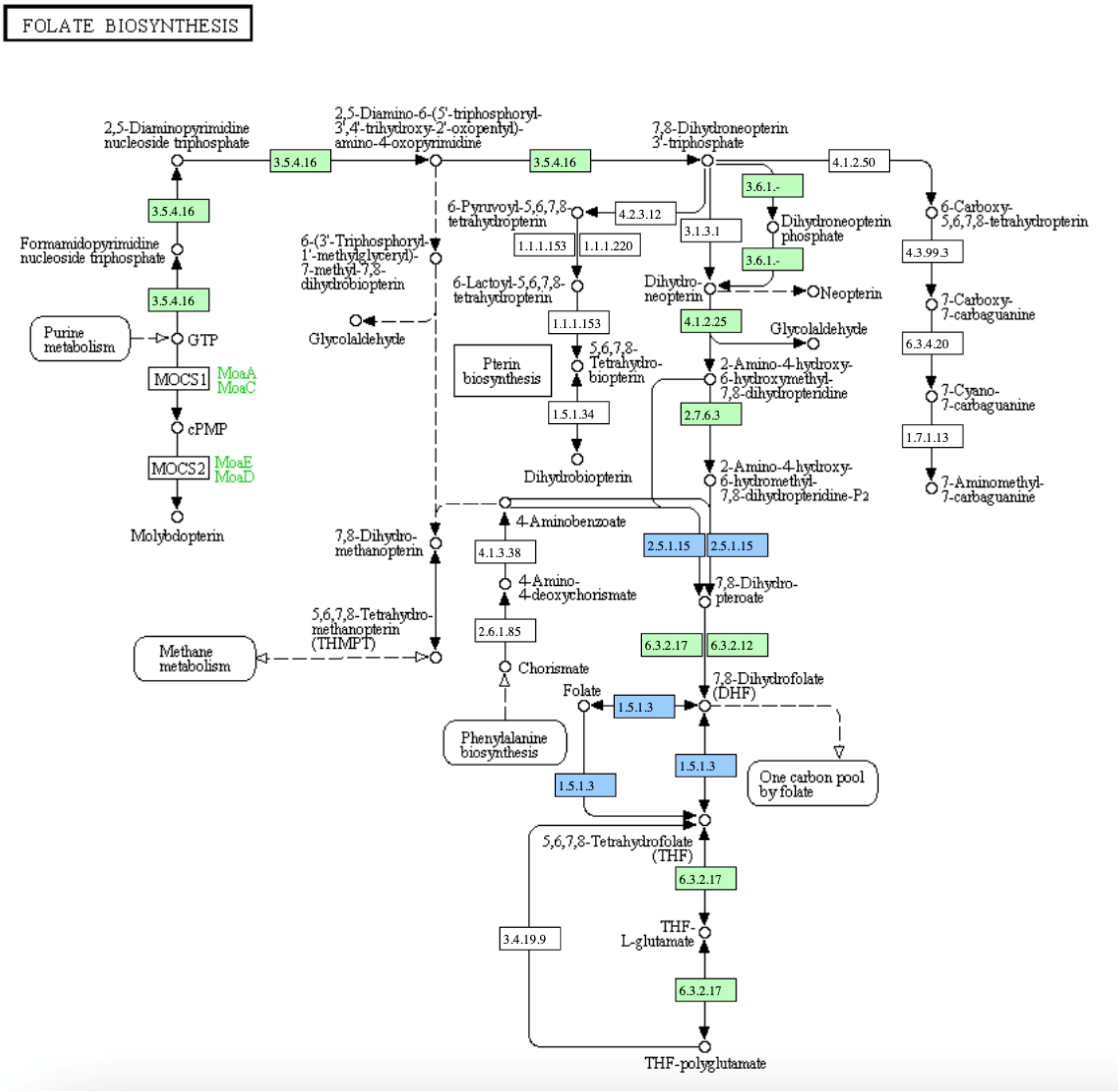

FOLATE BIOSYNTHESIS
2,5-Diaminopyrimidine nucleoside triphosphate
3.5.4.16
2,5-Diamino-6-(5'-triphosphoryl-3',4'-trihydroxy-2'-oxopentyl)-amino-4-oxopyrimidine
3.5.4.16
7,8-Dihydroneopterin 3'-triphosphate
4.1.2.50
3.6.1.-
6-Carboxy-5,6,7,8-tetrahydropterin
3.5.4.16
6-Pyruvoyl-5,6,7,8-tetrahydropterin
4.2.3.12
3.1.3.1
Dihydroneopterin phosphate
Formamidopyrimidine nucleoside triphosphate
6-(3'-Triphosphoryl-1'-methylglyceryl)-7-methyl-7,8-dihydrobiopterin
1.1.1.153
1.1.1.220
3.6.1.-
4.3.99.3
6-Lactoyl-5,6,7,8-tetrahydropterin
Dihydro-neopterin
Neopterin
3.5.4.16
7-Carboxy-7-carbaguanine
Purine metabolism
GTP
Glycolaldehyde
1.1.1.153
4.1.2.25
Glycolaldehyde
6.3.4.20
MOCS1
MoaA
MoaC
Pterin biosynthesis
5,6,7,8-Tetrahydro-biopterin
2-Amino-4-hydroxy-6-hydroxymethyl-7,8-dihydropteridine
7-Cyano-7-carbaguanine
2.7.6.3
1.5.1.34
cPMP
1.7.1.13
MOCS2
MoaE
MoaD
Dihydrobiopterin
2-Amino-4-hydroxy-6-hydromethyl-7,8-dihydropteridine-P2
7-Aminomethyl-7-carbaguanine
4-Aminobenzoate
Molybdopterin
7,8-Dihydro-methanopterin
2.5.1.15
2.5.1.15
4.1.3.38
4-Amino-4-deoxychorismate
7,8-Dihydro-pteroate
5,6,7,8-Tetrahydro-methanopterin (THMPT)
2.6.1.85
Methane metabolism
6.3.2.17
6.3.2.12
Chorismate
7,8-Dihydrofolate (DHF)
Folate
1.5.1.3
Phenylalanine biosynthesis
1.5.1.3
One carbon pool by folate
1.5.1.3
5,6,7,8-Tetrahydrofolate (THF)
6.3.2.17
3.4.19.9
THF-L-glutamate
6.3.2.17
THF-polyglutamate

J.

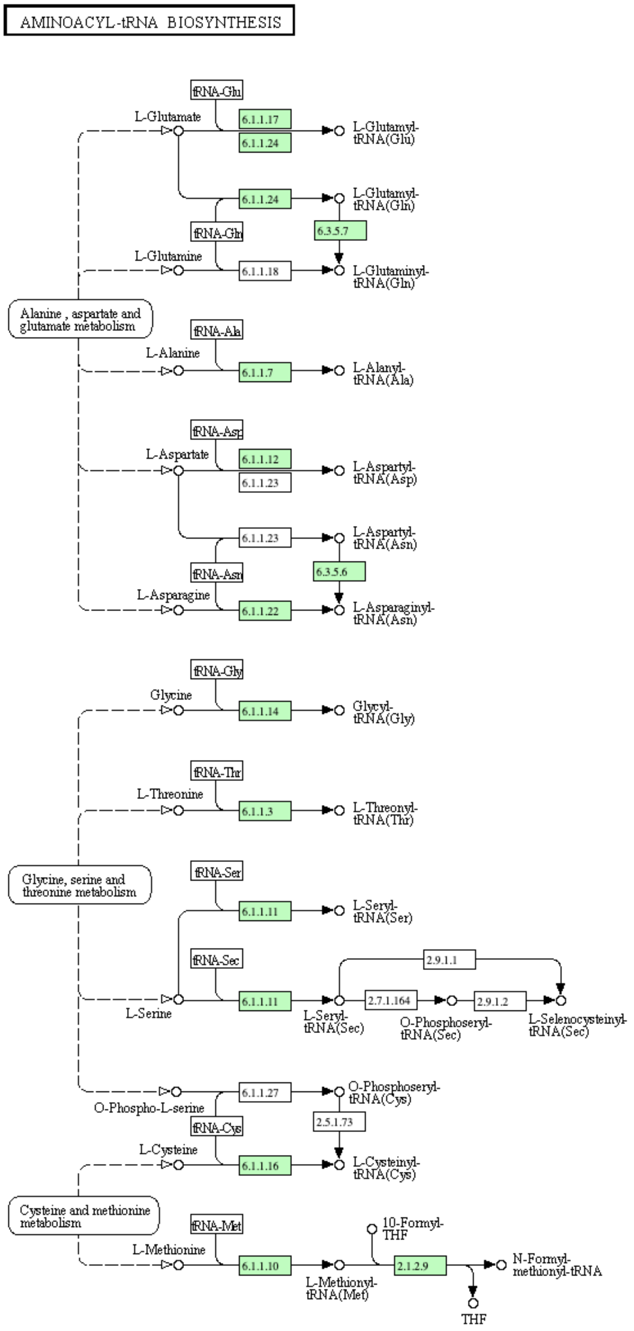

Figure S1: Pathways involved in the immune stress response and intestinal bowl diseases improvement. (A). Fatty acid biosynthesis (B). One carbon pool by Folate (C). Peptidoglycan Biosynthesis (D). Pyruvate metabolism (E). D-alanine metabolism (F). Amino sugar and nucleotide sugar metabolism (G). Biotin metabolism (H). Pyrimidine metabolism (I). Folate biosynthesis (J). Aminoacyl tRNA Biosynthesis.

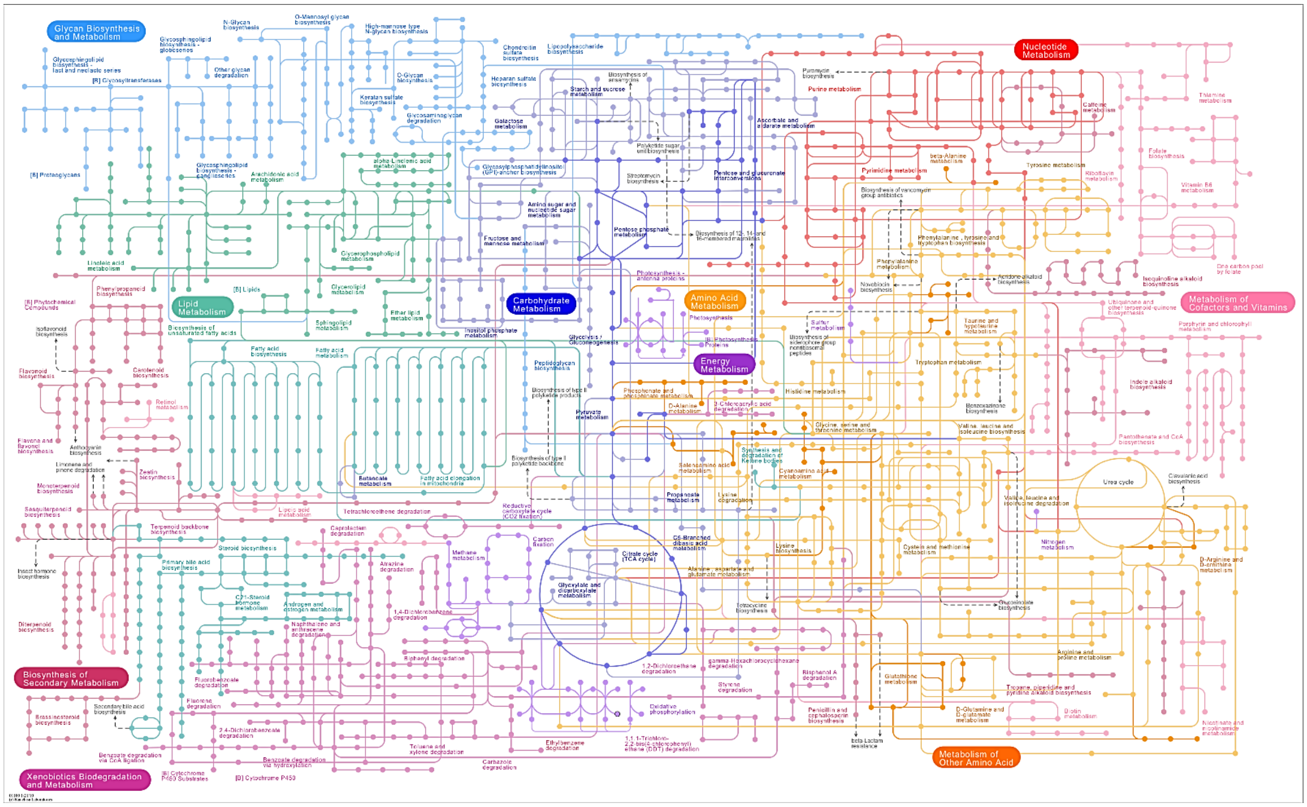

Figure S2: KEGG pathway for the synthesis of secondary metabolites and carbohydrates.

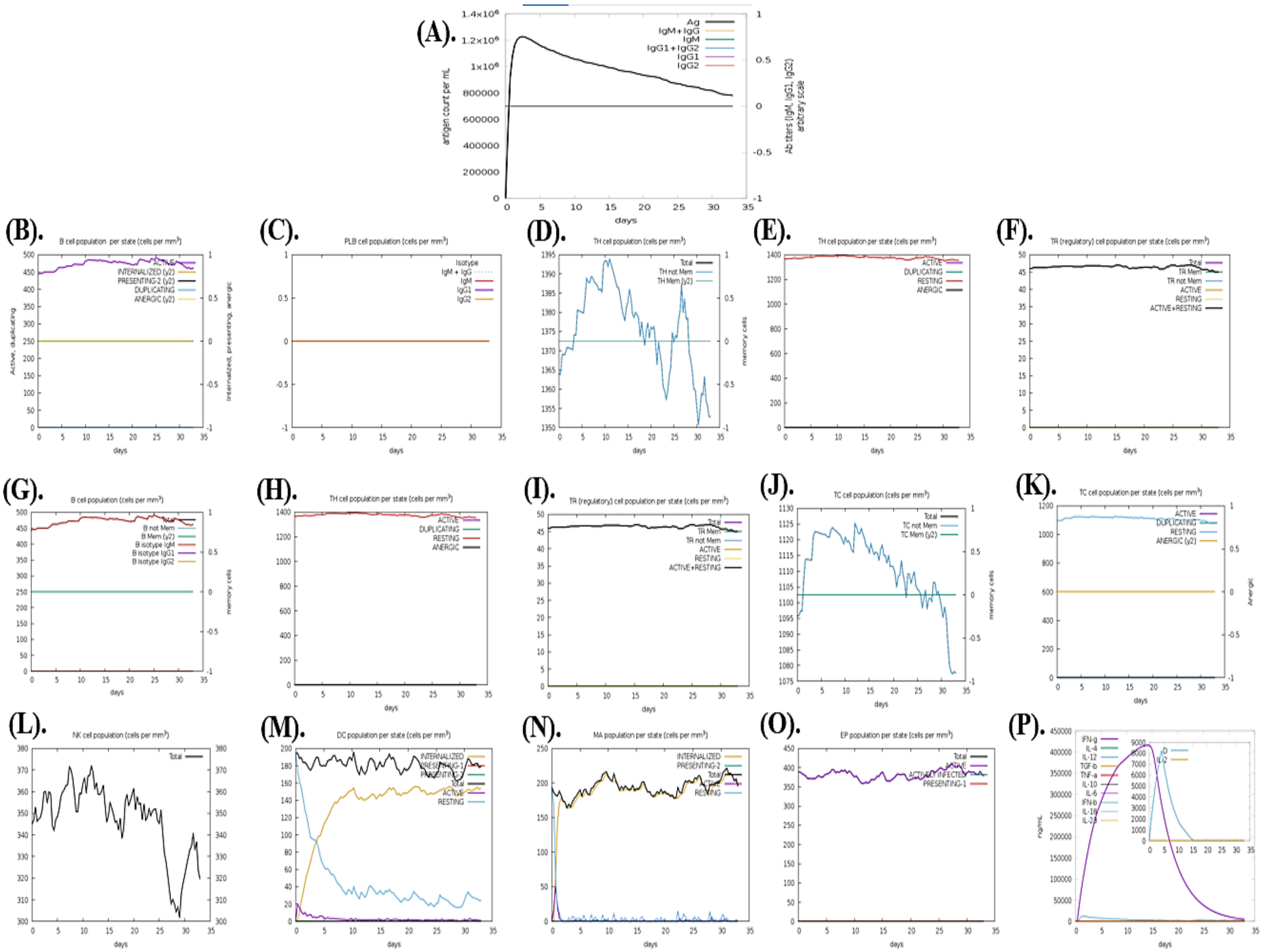

Figure S3. Immunomodulatory response after streptin dosage.

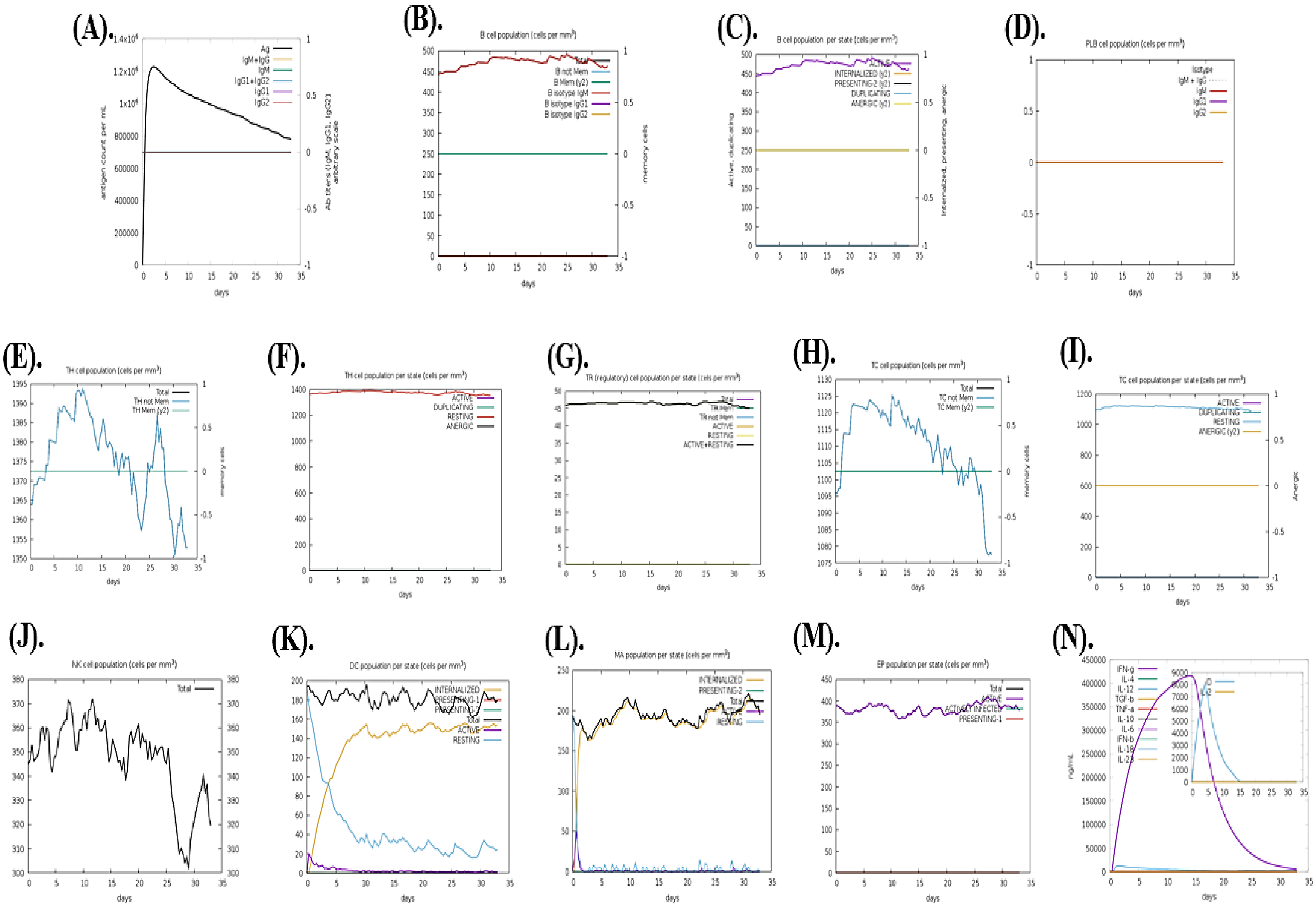

Figure S4. Immunomodulatory response after Ruminococcin-A dosage.
